# Patient views about the impact of ulcerative colitis and its management with drug treatment and surgery: a nested qualitative study within the CONSTRUCT trial

**DOI:** 10.1186/s12876-019-1085-y

**Published:** 2019-10-15

**Authors:** Frances Rapport, Clare Clement, Anne C. Seagrove, Laith Alrubaiy, Hayley A. Hutchings, John G. Williams

**Affiliations:** 1 Centre for Healthcare Resilience and Implementation Science, Australian Institute of Health Innovation, Macquarie University, Sydney, Australia; 2 Bristol Medical School, University of Bristol, Bristol, UK; 3 Swansea University Medical School, Swansea, UK

**Keywords:** Qualitative research, Patient, Ulcerative colitis, Ciclosporin, Infliximab, Surgery

## Abstract

**Background:**

A nested qualitative interview study within the CONSTRUCT trial was conducted to explore experiences and perceptions of patients with acute severe ulcerative colitis following treatment with infliximab or ciclosporin, surgery, or other medication.

**Methods:**

Two hundred seventy patients with steroid-resistant ulcerative colitis were randomised to either infliximab or ciclosporin. Interviews were conducted with 20 trial participants. Thirty-five data capture events took place in total, 20 interviews conducted 3 months after treatment and a further 15 interviews with the same cohort as second interviews at 12 months.

**Results:**

Disease duration varied but similar stories emerged about how people adjusted to living with ulcerative colitis. Issues raised by patients included; the debilitating effect of the disease on quality of life, living with the unpredictability of symptoms and treatment, dealing with embarrassment and stigma and the desire to share knowledge of the disease with others to combat the private nature of this debilitating illness and bring greater visibility to patient experience of symptoms and outcomes.

**Conclusion:**

Patients were more positive about treatment with infliximab than ciclosporin, mainly due to the cumbersome intravenous regimen required for ciclosporin. Prompt diagnosis is required and early reporting of changes in symptoms is encouraged to ensure appropriate treatment.

**Trial registration:**

This trial is registered with the ISRCTN registry; number ISRCTN22663589. The date of registration was 16/05/2008.

## Background

Ulcerative colitis (UC) is a chronic debilitating disease that affects ~ 150,000 people in the UK [1, 2]. There are many unanswered questions regarding the causes and course of the disease, its treatment, management, and outcome; despite a background of rapidly developing new therapies. Patients with acute severe colitis will require hospital admission. Treatment includes intravenous steroids but about 40% of patients are resistant to this [3, 4]. In the past, when no other treatment was available, emergency colectomy was the only option. Although mortality following emergency colectomy has fallen over time, 10% of patients die within 3 months of surgery [5]. Infliximab and ciclosporin are immunosuppressive drugs that offer hope for the treatment of steroid resistant UC, with evidence that they are both effective in the short term [6, 7], particularly among patients who respond partially to steroid treatment.

This article describes a qualitative study undertaken as part of a trial: Comparison Of iNfliximab and ciclosporin in STeroid Resistant Ulcerative Colitis: a Trial (CONSTRUCT), a UK-wide pragmatic randomised controlled trial (RCT) comparing the clinical and cost effectiveness of infliximab and ciclosporin for patients with steroid resistant UC, using quantitative, qualitative, health economic and data-linkage research methods [8–10]. This article adheres to CONSORT guidelines for reporting parallel group randomised trials [11] where the information is pertinent to the qualitative study being presented. The CONSTRUCT trial as a whole has been reported with adherence to CONSORT guidelines elsewhere [8, 9].

CONSTRUCT’s primary outcome measure was patients’ quality of life and the trial showed no significant differences between the two treatments in this measure of clinical effectiveness [8]. The qualitative element of the study contributed to the trial’s specific objectives of comparing quality of life across the two treatment groups from the patient perspective, investigating participants’ views about the two treatments, experiences of the drug regimens and opinions about how easy each drug was to handle, including perceptions of side effects and ongoing quality of life. The main aim of the qualitative study was to investigate patients’ priorities for their health and wellbeing, views of different drug treatment and surgery, and opinion regarding how well they responded to treatment.

Whilst studies have acknowledged specific characteristics of UC such as fatigue [12–15]; unpredictability [16, 17]; stigma [18] and surgical treatment [19, 20], to the best of the authors’ knowledge, no studies, qualitative or otherwise, have reported, in depth and across a wide UK-based patient cohort on adult patient experience of UC in relation to infliximab, ciclosporin, or surgical intervention. However, qualitative studies have considered: patient experience in relation to non-medical adaptation and non-medical intervention [21], disease burden [22], and a full review has been undertaken of generic meanings around living with inflammatory disease [23]. In addition, some qualitative studies have been undertaken relating to adolescent experience and those transitioning to early adulthood [24, 25], such as a study of adolescent hospitalization, and paediatric-provider relationships, as patients transition out of care settings. This gap is somewhat surprising given that qualitative methods are well suited to investigating personal experience in relation to drug taking or surgical intervention [26], and individual perceptions and belief and meaning systems [27, 28] and can help to clarify patients’ understanding of their disease and its treatment.

In order to determine and understand CONSTRUCT trial participants’ experiences and perceptions of living with UC and to disclose details of their experiences of two treatments with or without surgical intervention, the study team conducted telephone interviews at two stages in the study: at approximately 3 months, and again at 12 months after patients received treatment. This article presents a description of the interviews and outcomes following qualitative analyses of the interview data.

## Methods

### Design

Between June 2010 and February 2013, and with approval from a multicentre research ethics committee, CONSTRUCT recruited patients from 52 district general and teaching hospitals in England, Scotland and Wales. With informed consent, 270 patients, aged 18 and over, with acute severe UC, who failed to respond to intravenous steroids, were recruited to the trial and randomly allocated to treatment with infliximab or ciclosporin. The definition of acute severe ulcerative colitis used was patients admitted as emergency admissions with severe colitis [29], a Mayo Score of at least 2 (on endoscopic finding or clinical judgement) who had failed to respond to a course of about 2–5 days of intravenous hydrocortisone therapy, who also had either: histological diagnosis of ulcerative colitis in that episode; histological diagnosis of indeterminate colitis in that episode when clinical judgement suggested a diagnosis of ulcerative colitis rather than Crohn’s disease; symptoms typical of ulcerative colitis awaiting histology; or history of ulcerative colitis confirmed histologically. Patients did not choose which treatment they received or whether they had surgery. After consenting to participate in the trial they were randomly allocated to receive infliximab or ciclosporin. Surgery was one of the predefined outcomes measured in the trial and was offered as part of patient care if recommended by the clinical team. At the time of consent to the trial, participants were asked to consent to be interviewed at approximately 3 months and again at 12 months post allocation.

For this qualitative study there were no exclusion criteria. We purposively recruited equal numbers of participants from the ciclosporin and infliximab groups who had participated in the original trial and randomly received either infliximab or ciclosporin (baseline characteristics of the participants can be found in Table 1). Detailed inclusion and exclusion criteria for the trial and more extensive detail about all aspects of study design, data collection, analysis and reporting can be found in the full study report [9].

**Table 1:** Demographic characteristics of interview participants

Patient characteristic	Group a	Group b
*Gender*		
Male	7	7
Female	3	3
*Ethnicity*		
White	8	10
Asian or Asian British	2	0
Black or Black British	0	0
Other	0	0
Mixed	0	0
*Weight (mean, kg)*	72.7 (range 52 to 102)	76.9 (range 53 to 125)
*Height (mean, m)*	1.68 (range 1.55 to 1.79)	1.71 (range 1.58 to 1.80)
*Smoking Status*		
Non-smoker/ Ex-smoker	6	5
Never smoker	4	5
Smoker	0	0
*Truelove & Witt’s score*		
Severe	9	5
Not severe	1	5
*Median Mayo Score*	3	3
*Family History*		
Yes	2	2
No	8	8
*Quality of life scores*		
EQ 5D	0.38 (range − 0.08 to 0.73)	0.56 (range 0.29 to 0.81)
CCQ-32 (mean)	0.35 (0.13 to 0.56)	0.37 (range 0.24 to 0.49)

As 52 hospitals in the UK took part in CONSTRUCT, the trial participants were geographically widely dispersed, so for participant convenience and confidentiality, semi-structured interviews [30] were conducted by telephone when the patients were at home. Semi-structured interviews are a useful approach if a researcher wishes to ensure research participants have the time and space to expand on their answers narratively. Using this approach, participants were encouraged to explain any nuance or ambiguity in their initial answers and could expand, safe in the knowledge that they would not be interrupted. Using a telephone interview technique ensured wider data capture and greater reach and scope than would have been achievable had face-to-face interviews been attempted. Travel pressures and the accompanying resource implications were removed as was the potential for patients to get embarrassed discussing sensitive topics with a stranger, face-to-face [31].

Semi-structured interviews enabled the trial qualitative researcher to guide the interviewees through the questions whilst giving participants the opportunity to not only develop their responses but also raise issues that were important to them. In accordance with the qualitative study aims, the researcher explored participants’ priorities for their health and wellbeing, the ease with which patients were able to take the drugs and drug experience including their view of a drug’s side effects, and their response to treatment including the possibility of having to undergo surgery following drug treatments. An interview schedule was developed comprising key questions aligned to these aims. The opening questions were designed to encourage participants to think and talk about their health, what was important to them, and what good and poor health meant to them. These questions were followed by more specific questions about their treatment experiences, drug regimens, management of drugs and outcomes.

After the interviews commenced, it became apparent that some of the participants that had required surgery to treat their UC had had that surgery, between randomisation and their three-month follow-up and while the interview schedule was originally designed to examine overall views of surgical intervention, should it be necessary, the interview schedule was extended to capture the views of these participants as well.

### Sample and recruitment

The CONSTRUCT Protocol [10] defined a sample of 10% of the total of 480 randomised participants to be interviewed. Purposive quota sampling supported the identification of 12 consenting patients from each arm of the trial in view of the belief that a total of 24 participants, interviewed on two occasions (*n* = 48 data capture events), would yield a range of views and opinions regarding therapy and surgical intervention, as well as a mix of participants according to age and gender and patients from a range of centres. The sample size was supported by the literature’s response to sample size adequacy in qualitative research studies [32] and the need to *‘represent the voice of the people’*, with the aim of achieving data saturation at this point, or continuing sampling until data saturation was achieved (where no new data codes and categories are evident) [33]. As interviews would inherently disclose which trial arm the participant was in, the interviewers and analysts were unable to remain blinded. However, this would enable exploration of experiences of the drugs in depth, to meet the qualitative study aims and thus was deemed acceptable. Any potential bias in interpretation would be addressed by having more than one person analyse the data and come together as a group for consensus [34].

### Analysis

Data were analysed using both thematic and schema analyses and in accordance with the interview schedule [35]. Thematic analysis is an approach to analysis that implies both implicit and explicit ideas evident in the data collected, through the coding and classification of data into meaningful and relevant categories and themes. These categories and themes, as they are developed and refined, link the raw data to ‘summary markers’ that indicate the raw data’s main content and context. As a result, the thematic analytic framework needs to be both comprehensive and succinct, encapsulating the main issues arising in the raw material in an abbreviated fashion that is both accepted and clear [36]. Schema analysis, on the other hand, concentrates on identifying patterns within text which can provide valuable information about how people work, behave or communicate, concentrating on these speech patterns in relation to the original study aims and objectives [37]. Schema analysis presents these patterns as people’s approach to the use of cognitive simplification. It examines through speech patterns how people express their views, opinion and experiences and how they make sense of complex information to which they are exposed. Analysis starts with a thorough reading and rereading of pieces of text, such as interview or focus group transcripts, followed by the identification of key issues evident as themes and categories within the text. At the same time schema analysis looks at the repetitive aspects of patterns of speech and at the way people introduce metaphor to present themselves to others [37].

The thematic analysis commenced after four interviews had been completed and continued iteratively until data saturation was confirmed to have been reached [33] and where no new codes or themes were revealed. Due to the rich and detailed amount of data arising from the interviews we also deemed that ‘information power’ was sufficient to meet the study aims and objectives [24]. The transcripts were distributed to three qualitative researchers, one of whom was the primary researcher involved in data capture. Each researcher read the transcripts and considered the themes that were emerging. The three researchers then met to discuss their findings and develop an analysis framework and an iterative process followed with all the transcripts, whereby themes and concomitant categories were honed down and clarified amongst all three researchers through consensus agreement.

The wider CONSTRUCT study team was then involved in a schema analysis exercise [38]. This process was chosen for four reasons: a) to enhance the thematic analysis framework by verifying that all framework elements were cogent and comprehensible, b) to be multidisciplinary, c) to build consensus into the data assessment process to ensure decision-making and interpretation was rigorous and trustworthy, and c) to give those involved with the analysis of other study datasets an insight into the lives of the participant group. Members of the study team, a multi-disciplinary group of gastroenterologists, researchers, statisticians and trialists were asked to read three participant transcripts, one from a participant treated with infliximab, one from a participant treated with ciclosporin and one from a participant who had had ciclosporin followed by surgery. The members involved were then asked to write one-page schematic overviews of the main features of each of the three texts, in keeping with participants’ health and illness stories, and as they related to quality of life and drug regime issues. These overviews were then reviewed and integrated into one overview of each interview which was then discussed and refined in group discussions between team members. Data analysis followed a similar pathway to the team’s previous, qualitative, gastroenterology trials data, including secondary assessment by a team of qualitative data analysts of a subset of all interview transcripts to ensure the thematic template was rigorous and findings efficacious. As teamwork continued, combined analyses or all transcript schemas contributed to a final thematic framework [39].

## Results

### Number of interviews conducted

Whilst 24 participant interviews were planned, after 20 three-month interviews had been analysed, data saturation was reached, with multiple qualitative analyst coming up with the same themes and no new themes arising, and it was agreed that no further interviews were needed for the first stage of data capture. The 20 interviews at 3 months were split evenly between those randomised to infliximab and those to ciclosporin, and three in each group had also had a colectomy. There were three females and seven males in each treatment group and their ages ranged from 21 to 75 years, as shown in Table 2.

**Table 2:** Details of interview participants

	Infliximab		Ciclosporin	
	Males	Females	Males	Females
Number interviewed	7	3	7	3
Age range	23–64	21–44	27–75	31–59
Mean age	44	32	51	43

We conducted 35 interviews with 20 participants. The full cohort were approached for a second interview at 12 months and at this stage only 15 of the interviewees from the original cohort of 20 agreed to a second interview (eight infliximab, seven ciclosporin patients). The reduction in numbers resulted from: one participant having died, two participants not wishing to take part and our inability to contact two participants. From each of the two treatment groups, one participant (one male and one female), had undergone a colectomy, while one male participant who had undergone a colectomy before the first interview had since had two further operations (pouch surgery followed by ileostomy closure).

The length of time since the participants had been diagnosed with UC varied from just a few weeks to as long as 30 years. However, those who had only recently been diagnosed had generally been experiencing symptoms for several months. The demographic information for those patients who agreed to be interviewed was compared to the cohort of patients who were not interviewed and found to be comparable.

With many participants working, the interviews often took place when people returned home from work. The interviews lasted 30–45 min on average, and all participants agreed that the interviews could be recorded. Transcribed interviews were reviewed by the primary qualitative researcher before being anonymised by using the participant’s study ID, with all names and geographical details removed.

### Results of the interview analysis

Despite a wide age range amongst participants, and variation in the duration of their disease, similar stories were revealed about living and adjusting to UC, the physical, mental and emotional impact of the condition, the treatments, and people’s concerns and hopes for the future. The details of the participants’ experiences of UC are presented in a narrative form to reflect participants’ descriptions of how they ‘journeyed’ through their illness, and according to their individual storylines, which they generally presented as: realisation of the problem, receipt of treatment, management of the condition and views of what the future held. Presenting the findings in this way indicates the joined-up story of an illness course experienced as chronic, in terms of how UC becomes present and begins to take root in people’s lives, as well as people’s ongoing expectations and the consequences of the illness in the longer-term.

Similar findings from the interviews at 3 and 12 months are not reported to avoid repetition, but new views and experiences revealed at the 12-month interviews are included. By presenting outcomes in this way, we have aimed to identify both personal experience of health and illness, and opinions of care and treatment options from an individualistic perspective and over a period of 1 year. Data capture and analysis extended over the year and was finalised early 2016.

All quotes are from trial participants. Quotations are presented in italics followed by an anonymised code for each participant and the line number of the quote from the transcript.

### Participants’ views on their general health

For many of the participants, UC was the only significant illness they had experienced, and even for those with longer term UC, their recent inpatient stay was often their first admission to hospital with the disease. Thus, it soon became apparent that many participants had managed their problems themselves at home, and had not given much thought to the implications of ill-health, or needing to enter more formal healthcare facilities. It is against this background that participants reflected on their desire to continue to lead a ‘normal’ life, to retain the ‘freedoms’ to which they were accustomed, and to have the energy to undertake everyday activities including socialising, working and travelling, without constraint. They considered good health as having plenty of energy, not being reliant on medication, being symptom- and pain-free and not having to go to the toilet every few minutes. In contrast, participants related bad health to being ‘imprisoned’, that is to say, housebound, unable to socialise and to be burdened with physical constraints relating to pain, discomfort, lack of energy and a dependency on others.

These issues were raised on a number of occasions, especially when participants were asked how their UC affected their quality of life. Here the intensity of the suffering caused by UC became strongly apparent, particularly for those who had experienced symptoms for only a few weeks or months: *“a bit of a bolt out of the blue” (GHI0028.164)*. For this latter group in particular, admission to hospital was a shock and a frightening experience, and 3 months after their treatment they were still adjusting to this life-changing event.

At 12 months, while the course of the disease for the participants had differed, their basic desire to live a normal life, to be able to socialise, work and travel, had not changed.

### Onset of illness and diagnosis

A picture emerged of a convoluted and sometimes frustrating treatment story from early onset to diagnosis, via an often-misdiagnosed disease. The story frequently began with mild symptoms, such as going to the toilet more often and having some bleeding, which led to an eventual General Practitioner (GP) visit with a misdiagnosis of haemorrhoids: *“I wasted about a month going to see the doctor and he just gave me stuff for piles” (LMN0009.111)*. Some participants visited their GP several times, but it was only when their symptoms did not settle and became worse, particularly if weight-loss occurred, that they were referred for an endoscopy. Some participants who knew they had UC ‘soldiered on’ with their symptoms, and a couple of participants described feeling like ‘frauds’ as inpatients amongst some of the seriously ill people in nearby beds.

A range of issues were raised by participants who discussed not only their experience of taking drugs, but also their ability to control symptoms and manage medication and the impact of medication on symptom control. Some participants found medication control adapted well to their changing physical state while others experienced difficulties in attempting to manage UC. For this latter group, a range of different medications had been tested with unpredictable results over time. Of the full cohort, the participants who fared the best in recognising signs and symptoms, management of drugs and appreciation of different treatment options were those who knew they had UC before their admission. These participants were cognisant of the fact that as time progressed, treatment options narrowed to the more powerful drugs or surgery, which was still seen as the final resort.

### Unpredictability of the disease

The unpredictability of the course of the disease was a problem for many who had no idea when their next flare-up might occur. A flare-up resulted in pain and discomfort, increased and urgent bowel movements and bloody diarrhoea, necessitating frequent, rushed visits to the toilet. Participants described this loss of bodily function as being unable to rely on their bodies, leading to a sense of giving up control over their bodies. Life was planned around access to toilets amid the fear of having an ‘accident’, and participants jokingly commented on their knowledge of all the toilet stops on a regular car journey.

If a flare-up was not controlled, symptoms such as frequent toilet use – maybe 20 to 30 times during the day and night, had a massive impact on their lives, leading to severe quality of life deterioration within a matter of weeks. Participants described how normal life ceased to be manageable very quickly and how the unpredictability of the disease had a knock-on effect on their ability or desire to pursue everyday activities which had previously been a major part of their lives. When this was the case, all other activities had to be curtailed, in order for daily lives to be manageable: *“Colitis can make you a prisoner that you don’t venture far away from a toilet” (DEF0016.60)*.

At 12 months, all participants continued to be followed up at hospital and were on medication for UC; six patients reported no further symptoms, whilst three had experienced flare-ups. They understood that if symptoms developed, a prompt change in medication could prevent them becoming as ill as they had been before, and that they would need to contact their inflammatory bowel disease (IBD) nurse or GP for advice as they: *“would want to catch it earlier” (BCD0012.214).*

### Impact of symptoms on the life course

The impact of symptoms like diarrhoea and disturbed nights resulted in people’s health deteriorating. This resulted in reduced activities, and impacted socialising and working arrangements, to a state that people found themselves, in effect, housebound. During these phases, participants described suffering from a lack of energy, weight loss and exhaustion, the latter being something that many people emphasised several times during the interview: *“basically tiredness was one of the things that was really difficult” NOP0004.91)*. For many participants, life had to be put on hold; engendering a significant change which they found difficult to cope with: *“Flare-up – it was dictating to me what I was able to do, not me dictating it” (RST0021.15)*.

Those in employment described how they had tried to continue working for as long as possible until they reached a point where it was simply physically impossible for them to carry on. An issue for some younger UC participants was the potential detrimental effect on their careers, as a result of ongoing flare-ups requiring time off work.

The condition could, therefore, be seen to cause huge changes in lifestyle for those who had to take time off work, who could not venture out to socialise, and for those with young children who were unable to care for them properly.

There appears to be a trajectory for participants of any age of debilitation, in that they change from someone with an active lifestyle, to someone for whom the disease has an increasingly detrimental effect, which often leads to a state, that participants describe, in both mental and emotional terms, as reducing possibilities in their lives. This effects: going to the toilet, being in great pain and being bored but unable to do anything to alleviate the boredom:

*“I was spending all day in a chair or in bed barely able to eat and my life just consisted of going to the toilet and sitting still trying to occupy my mind and not go crazy.” (GHI0028.19)*

For some patients, UC took them entirely by surprise, changing them from someone with good health to someone with a chronic condition. For many, life took a different and unexpected path, where their freedom to travel, pursue hobbies and to work was curtailed or restricted because they could not rely on their body functioning properly. For those wishing to travel, there was the added difficulty of obtaining travel insurance.

During the 12-month interviews, the quality of life for participants who managed to bring their symptoms under control, had improved, with some even able to plan events with confidence. Participants spoke of the impact of extreme illness, and being diagnosed with a chronic disease, leading directly to significant life changes, affecting their jobs, precipitating a house move, or perhaps leading to self-employment.

### Embarrassment about sharing knowledge with others

In contrast to some other chronic conditions, many UC sufferers considered it to be an embarrassing illness because of its association with ‘bottoms’, ‘bleeding from your bottom’, diarrhoea and going to the toilet. As a result, participants explained that they were selective about who they shared information about their disease with, often only discussing it with close family members and friends, rather than with acquaintances or work colleagues. However, when informed, employers and work colleagues were often found to be understanding and supportive.

UC is a ‘secret’ disease, difficult to discuss with few clear and recognisable manifestations. In addition, manifestations change rapidly and this unpredictability can lead to problems in treatment planning and therapeutic discussion. Participants in this study noted that others were often unaware of what the experience of UC was like for them, particularly members of the wider public implying a lack of a broader awareness of UC as a chronic condition. Participants, including those who had had surgery, remarked that a lack of public interest and awareness was exacerbated by little media interest, few reviews of the disease, and rare media discussions about the profile of UC compared to other chronic conditions. Participants noted that were famous people to come forward more frequently, who had the disease, more media interest might be engendered. The low profile was attributed to the embarrassing aspects of the disease which meant it was perceived differently to other chronic conditions.

At 12 months, similar issues arose, but a participant reflected: *“that you need to be able to speak to people and share what’s happened to you” (TUV0001.584)*, while two surgery participants, in particular, were keen to share their experiences with other sufferers and help them understand the impact of surgery and coping with a stoma.

### Impact of UC on family and friends

The life-changing nature of UC clearly impacts on families and friends, particularly during periods of flare up. Those with young children found themselves unable to look after and play with their children, while others found it affected their partner’s social life and could affect family travel plans:

*“ … would like to go around the world with my wife and we can afford it now but I simply, at the moment haven’t really got the energy for it” (ABC0010.86)*.

However, the predominant observation made by participants was that the support provided by family and friends was invaluable, in both practical and emotional terms. Ensuring others could support them was a positive outcome, and helped patients to cope mentally, as the condition became more difficult to manage.

At 12 months, the impact of UC on family and friends was spoken about by those who had had surgery. A participant who visited his workplace to discuss returning to work was aware that word had got out that he had a stoma and found some individuals did not look him in the eye easily but were: *“constantly staring to see if they could see this thing” (DEF0016.680)*. He found this rude, and his wife was so annoyed that she had to walk away. However, one participant felt that friends and family got bored with the disease so talking to the interviewer was helpful: *“talking to an absolute stranger, vague stranger, there’s something quite soothing about it … just go through it from start to finish” (MNO0034.729).*

### Acquiring knowledge about ulcerative colitis

Participants appeared to be well-informed about their condition, using the internet to find out more and turning to support group literature, such as documentation provided by ‘Crohn’s and Colitis UK’ (a national support organisation). In addition, many patients were in contact with an IBD nurse at their local hospital, from whom they received information and help when their symptoms exacerbated. However, participants commented on the lack of availability of an IBD nurse at all hospitals and were of the opinion that their support would be invaluable, and their role should be introduced more thoroughly.

Despite information available, participants had a number of unanswered questions surrounding the cause of their UC. They spoke about the possible genetic links to their disease and mentioned the need for more research in the area, in order to ensure that other family members could be supported if they did get UC or could be prevented from suffering with the disease. Participants were also unsure about what triggered a flare–up and wanted more information on this particular aspect of their disease, including information about the links between UC, stress and diet. Ultimately, participants wanted to see a cure for the condition; and whilst recognising that it might be too late for them, they wished to support others from having to suffer with the disease.

Participants appeared to be less questioning about UC at 12 months, as only a few comments arose about the cause and cure. However, the suggestion that they had easy access to care appeared to be more important to them at this point in their disease.

### Shared treatment decisions

Participants were well-informed about their UC, knew the signs and symptoms that indicated a flare-up and the various treatments available to them, and with this knowledge, they saw themselves as taking part in shared decision-making about their treatment with clinicians. This was particularly evident with regards to surgery as an option, when participants clearly understood that they had failed to respond to the medical treatments:

*“ … spoke long and hard to the surgeon and medical doctors and we all agreed that my quality of life would be improved if the bowel was gone” (DEF0016.362)*

Participants clearly understood that if they failed to respond to the various treatments, there could be a time when their body would make a treatment decision for, them rather than the decision being made by them or their clinician: *“if you are so bad and it gets to the point where it could put your life at risk then obviously you have to have it done” (JKL0033.521)*.

### Comparison of infliximab and ciclosporin from the participant perspective

All the participants except one had only ever experienced one of the drugs, (ciclosporin or infliximab) and were extremely ill when the treatment was first administered. As a consequence, whichever drug led to a participant’s health improving, was viewed positively, particularly if it meant that surgery could be avoided.

#### Ciclosporin

Participants found being hooked up to a drip continuously for several days at a time was problematic, in that it was restrictive: “*… loss of freedom, I found that quite irritating, personally found that frustrating” (BCD0012.218)*. It also meant being woken up at night for infusion bag-changes which caused anxiety, especially when a bag-change was delayed. Some found the infusion easier than the oral form of ciclosporin, as blood tests were required to monitor ciclosporin levels, tablets were large and had a distinctive smell, and participants had to remember to take tablets and have them with them when they went away. However, tablets meant ‘no needles’ and could be taken at home.

There were a number of side effects directly linked to ciclosporin, including mood swings, rashes on arms, tingling in fingers and toes, increased facial and body hair, tiredness, hand tremors and cramps in the hands and feet. Women in particular worried about facial hair during the period in which they took ciclosporin.

Two participants did not respond to intravenous ciclosporin and required emergency surgery. A third participant initially responded but relapsed and was given infliximab (the only participant to have experience of receiving both treatments). In the short term, those who did respond commented that they had avoided surgery, it got their condition under control and that they felt much better. At 3 months, participants found it difficult to comment on the impact of ciclosporin on their current health as most were no longer taking it and were on other medications.

#### Infliximab

Participants found the short intravenous infusion easy: *“I was just lying in a hospital bed so I could doze off, read a book” (KLM0010.153)*. They had the same opinion about further treatments that could be provided to them as an outpatient - appreciating the shorter time the treatment took and not having to take tablets: *“there is not any managing of it because it’s a two-hour infusion and then you’re not due back in for another six to eight weeks so in that way it’s fantastic” (OPQ0005.395)*.

The side effects of infliximab were more immediate, such as a ‘weird’ sensation in the legs, feeling vague and ‘dopey’ and becoming very hot during the infusion, and one participant described getting hot at night for two to 3 weeks following infusion.

Three participants did not respond to infliximab and required surgery. A participant who was switched to infliximab noticed a positive difference in her condition within a day. In the short term, those who responded well had positive comments to make, as the treatment meant they avoided surgery. They noticed a rapid difference in their state of health and viewed infliximab as a *‘miracle drug’ (GHT0034.331)*. Again, it was difficult for participants to comment on whether they felt the infliximab was still having an effect at 3 months as they were taking other medications. A couple of participants felt the effects were still lasting and two participants felt the effects wore off as they neared the time of their next infusion.

By the time of the second interview, none of the participants were receiving ciclosporin or infliximab. One participant from each group had experienced a flare-up whilst four infliximab and two ciclosporin participants had no further symptoms since completing the trial treatment.

At 12 months the majority of the participants spoke more about other treatments they had received since the trial treatment, but two recalled that ciclosporin had been a successful treatment. Three commented on the convenience of infliximab and that they would be happy to have it again, whilst one stated that treatment with infliximab: *“every so often would have been easy-peasy” (QRS028.342)* when compared with having surgery and living with a stoma. One participant explained that because of funding restrictions, her local primary care trust would not allow her to continue treatment with infliximab and she felt her: *“quality of life would be much better now if I was able to continue it” (MNO0034.780)*.

### Surgery

Surgery was considered a final option by most participants who took part in the interview study, who described surgery in terms of the fear and anxiety it raised in them. They used a range of terms to describe their worries, terminology that was particularly indicative of a sense of loss of a body part, such as ‘losing’ a colon. Participants were particularly nervous about the outcome of surgery and the possibility of having to wear a colostomy bag weighed heavily on their minds, as the following quotation indicates from one patient who was extremely concerned before their operation at the thought of having to get used to a colostomy bag, post-operations but whose fears were allayed when they ended up not needing one: “*… I walked out of there without a bag … it’s just the fear of the unknown* [having to manage with a colostomy bag]*, the effect it can have on your life I suppose, life changes” (TUV0001.242)*.

Nevertheless, for those who went through with surgery the end result was commonly unexpectedly positive, and most participants, in weighing up the consequences of surgical intervention, retained an open mind to the possibility of this being an ultimate outcome of their disease progression were drug or therapeutic intervention to fail. Those who were told that they would need to have surgery often described becoming resigned to the inevitability of the operation, accepting the next steps in their treatment: *“I felt really fed up so I was actually quite happy, to be honest to have the operation by the time it came round … it wasn’t really a difficult decision to make in the end” (GHI0028.207)*.

Surgery participants would have wanted to continue with medical treatment, but after surgery had taken place they expressed relief that it had been done, as it meant an end to their UC: “*… the moment I woke up after the operation I thought, ‘cor this is fantastic’ … the fog had cleared” (STU0008.188)*. However, one participant had fought against surgery when diagnosed 55 years before, and was relieved that he had not had the surgery as a young man, whilst another described feeling suicidal after surgery. Most participants viewed surgery as having been a good decision but stressed that it did create issues in relation to the practicalities of managing the stoma and the bag, restricting them to certain types of clothing and causing concern about how others would react to seeing the bag, thus curtailing activities such as swimming: *“I’ve got to keep my T-shirt on which is something I wouldn’t look forward to because I like to jump in the sea … I don’t want people staring at me all the time” (STU0008.155)*. Participants conveyed the point that being older made it a little easier to cope with the outcomes of surgery: “*… I think when you’re a bit older it is a lot easier to deal with, mentally really” (QRS0028.307).*

Some participants were selective about whom they told about surgery, because they felt people would not understand. They tended to tell family and close friends, and where necessary, work colleagues, and they generally found that people were supportive. However, some participants were clearly very conscious of the physical changes to their body and the impact on personal relationships, whilst partners were often said to be understanding and supportive.

Several participants spoke about the possibility of having surgery to restore bowel continuity and closure of their colectomy and age was shown to influence attitudes, with younger participants keen to get on with the process of getting life back to normal and older participants appearing more likely to accept life with the stoma: *“If I was, don’t know, 20 years younger, I’d probably go for reversal because I wouldn’t want to live with the bag” (QRS0028.289)*.

At the 12-month interview point participants who had had a colectomy remarked on strong ongoing personal relationships and little detrimental effect as a result of surgery. One participant, who had had surgery during his first admission, had gone on to have two further operations to achieve reversal following which he noted improved quality of life and felt no regrets. Two men had found that intimate relationships had suffered but these were an exception to the rule. Indeed, at 12 months many surgical patients considered surgery as a cure.

### Concerns, hopes and aspirations for the future

Participants who were yet to have surgery were worried about where their disease course might take them and what future treatment might hold for them. Recently diagnosed patients took time in their adjustment to the disease especially if they did not yet have corresponding signs and symptoms that led them to feel unwell or mentally vulnerable. The disease often precipitated a gradual physical and mental decline and consequently participants found it hard to perceive of a time when their bowel habits would deteriorate still further, and where the need for medication would be more pressing Nevertheless even for those people whose symptoms had resolved, taking medication was a constant reminder that: *‘it might come back’ (BCD0012.520&600).* Younger patients worried about the effects of the disease on long-term plans, including starting a family or sustaining relationships.

The 12-month follow-up interviews were a reminder to patients of how life had changed. Seven of the eight participants who had not needed surgery were coming to accept the challenges and limitations imposed by their illness and seemed more confident in recognising their treatment needs. They were reassured by ready access to advice and guidance from all specialist staff, including IBD nurses, but surgery remained a clear area of uncertainty. Participants had a range of questions still unanswered about: stoma reversal, impact of surgery on personal relationships, stoma management and others’ involvement in future care including close others needing to handle their stoma.

## Discussion

Given that CONSTRUCT’s primary outcome measure was patients’ quality of life and the trial showed no significant differences between the two treatments in this measure of clinical effectiveness [8, 9], the results of this qualitative study assume greater importance, and the validity of the findings require detailed scrutiny. The participants’ views about UC and treatment with infliximab, ciclosporin and surgery are important, unique and detailed, and were derived from a broad age range, and a UK-wide cohort of patients in the largest UC trial of its kind. The interviewees were chosen at random, but stratified by age, treatment and surgery, and the demographic and baseline characteristics of the 20 participants interviewed, 15 of whom were interviewed on two occasions, were no different from the rest of the participant population in the study. Applying the criteria of credibility, transferability, dependability and confirmability set out by Guba in assessing the rigour and trustworthiness of data [40], we believe we used a rigorous and valuable qualitative approach. This included checking that the data being collected and the analysis of that data was shaped by what the participant population was saying, ascertained by both building the qualitative dataset iteratively, so that comments made by one participant could be expanded and enriched by the comments of others, and by ensuring data analysis included extensive team work and cross comparisons. We have used this approach across a range of disease-types [39, 41], and it is dependent on clarification by multiple analysts, working together, that knowledge and understanding is agreed, and consensus has been reached.

The data from interviews reported in this article, with patients at both three and 12 months after being admitted to hospital with UC, highlights the immense physical and mental strain of the disease on people’s lives. This closely aligns with the findings of Rubin et al., who in their 2008 study describe the difficulties patients face in living with the debilitating disease and the struggles patients face managing long-term medication-taking [42]. Some aspects of this appear to set the suffering of people with UC apart from other chronic diseases, as a result of unpredictable symptoms and the urgency of needing a toilet. In addition, unique to UC, is the consensus view that this is an embarrassing disease, and thus difficult to share with others, along with the stigma associated with having a bowel problem. Taft et al. have defined UC as a long-term secret disease, leading to increased psychological distress and reduced quality of life, going so far as to describe it as the disease of body stigma as a result of the uniquely related bodily functions involved [43]. In this study, this clearly affected participants’ thoughts about their quality of life, relationships with others, plans for social and work life, and future aspirations for disease management.

Unique to this study are detailed views about the use and handling of two UC drugs, ciclosporin and infliximab, unlike other publications on patient experience of UC more generically focused on patient experience across disease symptoms and treatments [23]. All the participants bar one had only ever experienced one of the drugs, (ciclosporin or infliximab) and were extremely ill when the treatment was first administered. As a consequence, whichever drug the participant received was viewed positively, particularly if it meant that surgery could be avoided. However, ciclosporin was reported in a more negative light than infliximab across the cohort, particularly in terms of the length of time necessary for the intravenous infusion, as opposed to those receiving an infusion with infliximab, which was quick and straightforward. Participants being treated with ciclosporin also commented on the size of the oral medication, the problems surrounding having to take tablets on a daily basis, the inconvenience of having to have regular blood tests, and the long list of side effects linked to taking ciclosporin. The participants in this study who were allocated infliximab liked the fact that they only had to return every few weeks to clinic for ongoing treatment, and that they did not have to remember to take tablets every day. Participants noticed side effects with both treatments, but those with infliximab tended to be more immediate, and lasted for a shorter period of time. It is interesting to note the comments of the only participant who was able to compare the two treatments who mentioned that it was only after taking infliximab that they really began to feel better. The lack of comparative data between the two drugs may reflect the fact that in the UK, infliximab is restricted for use by some funding agencies (primary healthcare Trusts). This is confirmed to be the case by Crohn’s and Colitis UK, who in their online information sheet explain that *‘Infliximab is a very expensive drug, so hospitals have to apply for funding before they can use it on a patient and must review how effective it is from time to time’*. [44] Frustration at this was expressed in the results section of this paper by one participant (*MNO0034.780*) who felt that as a result of such restrictions, she had not been encouraged to continue her infliximab treatment.

The interviewees included a widely-based constituency in terms of disease diagnosis and consequent treatment pathways. There were those who had been diagnosed with UC many years ago, some of whom had suffered from the disease before diagnosis for an extended period of time, whilst others who had been diagnosed during their recent admission to hospital, had only had a short-lived experience of extreme symptoms. However, the severity of symptoms was something all participants had in common, which for many indicated how quickly health can deteriorate with UC, even leading to the need for a hospital admission.

The extent to which UC had impacted on quality of life for participants was clear because of the emphasis put on how highly they valued what they called their ‘normal’ life; their ability to work and socialise. Their normal life was taken away by the physical symptoms and these combined with being woken at night to use the toilet, caused severe fatigue, which has also been noted in other studies [12–15]. These symptoms had a significant impact on people’s normal life, and as their quality of life deteriorated and they became socially isolated, this affected their ability to work and made them feel even more isolated and inadequate.

Whilst studies acknowledge that UC is an unpredictable disease [16, 17], before the detailed reports of our participants, there was little evidence of exactly what this meant for patients, nor how the use of specific drugs and longer-term impact of drug-taking influenced views of UC. However, the interview data in this trial demonstrated that the issue of unpredictability: exactly when a flare-up might occur, whether treatment would be effective and for how long, as well as concern about soiling oneself, was significant and ever-present for people who had to work around the effects of symptoms when planning journeys. For others, projecting about the longer-term experiences of treatment management, and the physical difficulties of having to abide by long-term treatments and all they entailed led to despair. For some, changes of strategy to enable patients to live with the disease were needed and to counteract some of the most pressing worries, for example: changes in lifestyle, curtailment of holidays, stopping hobbies and attending sports events. Interestingly, unpredictability also extended to the way participants spoke about their treatment and whether it would work. For some, the need for eventual surgery was also uncertain and led to additional anxieties exacerbated by thoughts about outcomes of surgery and when it might be urgently required.

Although the stigma associated with UC has been described before [1] this qualitative study demonstrates participants’ feelings about the extent of the embarrassment people experience, and highlighted how this is not something people find they can readily speak about. Taft et al., in their study of stigma related to inflammatory bowel disease reported that stigma was a widespread phenomenon noting that from a patient cohort of 211 patients, 84% reported related stigma impacting quality of life, self-esteem, adherence to drug regimes, and self-efficacy [43]. Apart from concerns about accidents, participants in our study indicated their extensive worries about sharing information with family and friends or work acquaintances. Participants felt that a wider public understanding of the disease, which could be achieved via media channels, would alleviate some of the embarrassment factor, and help patients discuss the disease in more detail with others. A media awareness campaign could serve as an important way of raising awareness about UC (and Crohn’s disease), informing the general public of both the disease and about different surgical interventions and outcomes. The literature indicates that misunderstandings around UC and public knowledge of digestive health and related diseases can be traced back decades. In a 1987 publication by Kreps et al. [45], for example, a widescale survey of the American public’s knowledge of gastroenterological disorders and their impact identified an uninformed and misinformed general public, with little health promotional support, leading to minimal healthcare prevention strategies and poor self-management of care.

It was difficult for participants in this study to attribute their condition at three and 12 months to the trial treatment, as very few were still taking the trial drug (ciclosporin is not used in the longer term and there are funding issues with infliximab). However, if the drugs had been successful it was clear that they got the participants to the point where other medication could then maintain their remission.

In total, eight of the interviewees had had a colectomy and their data added to the evidence [19, 20] about patients’ experiences of surgery as a necessary and often essential treatment for UC. This qualitative study demonstrates that before surgery patients feared the unknown, and surgery was seen as a last resort, and then only when everything else had failed to save their colon was surgery a possibility. However, most patients were pleased with the results of surgery, and some expressed regret that it had not been carried out earlier. The unpredictability of UC has been emphasised in this study and for many having surgery suddenly removes the unpredictability of symptoms and medical treatment. However, after 12 months, difficulties of living with a stoma came to the fore for some people, leading to a deterioration in quality of life if the operation could not be reversed.

### Strengths and limitations

The results of this qualitative interview study demonstrate the importance of incorporating qualitative data within a clinical trial as this rich data clearly complements the study’s overall aims and objectives. In a clinical trial showing no significant difference in clinical effectiveness between the two drugs infliximab and ciclosporin, understanding patients’ views about their disease and its treatment becomes more important as patient views help inform disease management. The data collected in this study provides an in-depth view from a patient perspective, of the reality of living and adjusting to the disease, the degree to which the disease impacts on people’s lives, and the effects of treatment.

The study has its limitations, however; whilst qualitative research does not seek population representativeness, or provide generalisable findings, the number of interviews could be considered a study limitation and we cannot make claims for the whole of the UC patient population in the UK, in particular with regard to patient views about surgery as a treatment for UC. More research needs to be conducted to explore the views of UC patients, post-surgery, postulating to a global populous and in order to provide more information to aid the decision-making process of those facing surgery. As the views of participants following surgery was generally positive, there should be further research into the surgical treatment of UC as a real alternative to medical treatment.

## Conclusion

To conclude, this article is the first to report a qualitative study comparing infliximab and ciclosporin in relation to patient experience and the treatment of UC, from a patient perspective, and showing how patients treated with infliximab generally had a more positive outlook on treatment than those treated with ciclosporin.

The qualitative study covered a wide range of domains regarding the patient journey, confirming the degree, type and severity of the physical symptoms that patients with UC experience, particularly when suffering a flare-up, and illustrating some of the ways that people learned to live with the disease. However, importantly, these data show the wider effects of these symptoms on patients’ lives and indicate how easily people can become socially isolated and stigmatized as their health deteriorates and symptoms become more extreme. Consequently, we conclude that greater efforts should be made to ensure prompt diagnosis, to encourage known UC patients to report changes in their symptoms, and for appropriate treatment to be given as quickly as possible, sensitive to the needs of patients and the possibility that not only symptoms, but also personal experiences can rapidly change.

A significant issue that emerged was that UC and its treatment remain a significant burden on patients’ lives because of its unpredictability and lack of knowledge surrounding the disease and we recommend that better support and greater information should be considered a priority, to enable patients to manage the impact of their symptoms and ongoing treatment regimes, more effectively. Greater information should also be available for the general public and a new, and concerted effort made to improve healthcare promotion strategies to increase disease-knowledge.

Surgery has been shown as a positive option for many patients. In light of this, further research is needed investigating the implications of surgical treatment of UC, as a real alternative to medical treatment, in line with patients’ views, post-surgery, to support the decision-making process of those facing surgery.

Finally, an awareness raising campaign about UC (and Crohn’s disease), including details about surgery, would encourage people to seek help earlier and help to destigmatise UC, thereby reducing the embarrassment felt by sufferers and those living with the outcomes of surgery.

## Data Availability

The data that support the findings of this study are available from the corresponding author, Frances Rapport, upon reasonable request. The data are not publicly available in order to protect patient privacy.

## Abbreviations

CONSTRUCT: Comparison Of iNfliximab and ciclosporin in STeroid Resistant Ulcerative Colitis
GP: General practitioner
IBD: Inflammatory bowel disease
RCT: Randomised controlled trial
UC: Ulcerative colitis
